# Novel technique for repair of symmastia using external negative pressure therapy for continuous pre-sternal compression

**DOI:** 10.1093/jscr/rjaf006

**Published:** 2025-01-31

**Authors:** Zachary Lawrence, Joel C Stroman, Heather Karu

**Affiliations:** Department of Surgery, University of South Dakota Sanford School of Medicine, 1400 W. 22^nd^ St, Sioux Falls, SD 57105, United States; University of South Dakota Sanford School of Medicine, 414 E. Clark St, Vermillion, SD 57069, United States; Department of Plastic and Reconstructive Surgery, University of South Dakota Sanford School of Medicine, 1500 W. 22^nd^ St Suite 101, Sioux Falls, SD 57105, United States

**Keywords:** symmastia, breast surgery, techniques

## Abstract

Symmastia, a term used to describe the medial intermammary confluence that may appear as a complication following breast augmentation or reconstruction, is a cosmetic issue breast surgeons occasionally encounter and must be prepared to correct. Over-dissection of medial structures, oversized implants, and forces applied to implants in the sub-pectoral space have all been proposed as potential causes of the development of symmastia. Numerous techniques to correct symmastia have been described in previous literature, including capsulorrhaphy, dermo-sternal adhesion, neo-pocket creation, and techniques based on muscle repair. However, these methods are susceptible to recurrence. In this case report series, we present three cases in which symmastia was successfully corrected following surgical repair with post-operative external negative pressure therapy over the intermammary sulcus.

## Introduction

First formally described in the mid-1980s, the Greek term “symmastia” (or synmastia) was proposed to denote the phenomenon where medial (intermammary) confluence exists between the left and right breast due to the presence of an aberrant pre-sternal web of skin and soft tissue, thus obliterating the characteristic architecture of the intermammary sulcus [1]. Several techniques for correction of symmastia have been described over the past two decades, including the neosubpectoral pocket repair, capsulorrhaphy, anchoring of dermis to sternal periosteum, use of an acellular dermal matrix (ADM) sling, and delayed secondary implant reconstruction (with or without relocation to a new tissue plane)–all of which are associated with high recurrence [2–5]. In this three patient case report series, we demonstrate the novel use of external negative pressure therapy following surgical repair of symmastia as an adjunctive tool to reduce recurrence.

## Case series

**Figure 1:**
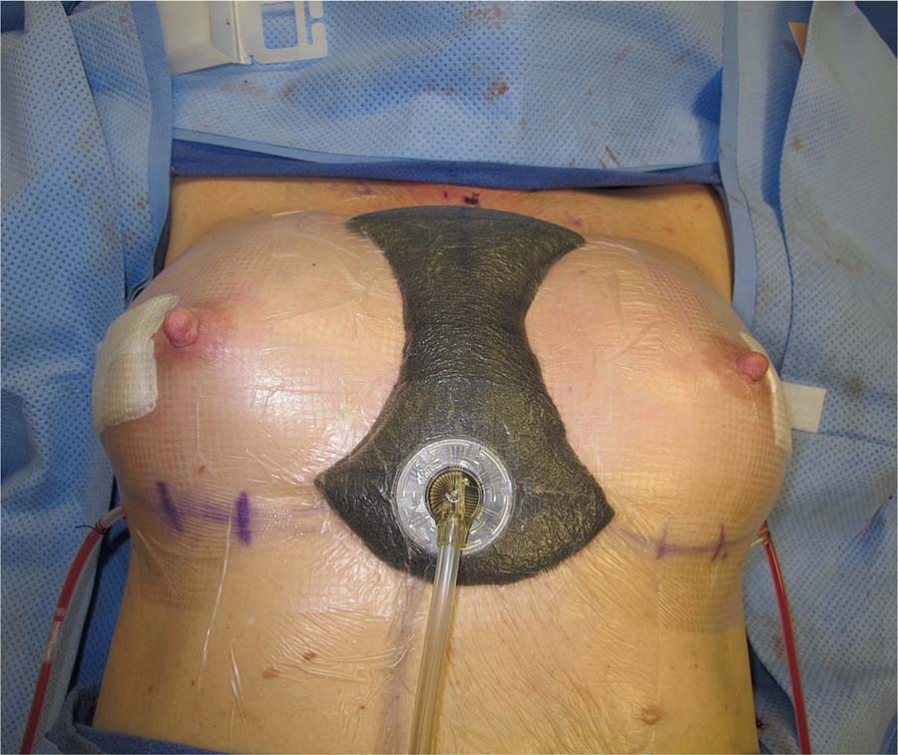
Photo of negative pressure wound device compressing the intermammary sulcus after surgical correction of symmastia.

The first patient is a 71-year-old BRCA2 positive female who developed symmastia following bilateral prophylactic mastectomy in 2010 with staged sub-pectoral implant reconstruction. Her symmastia recurred after an initial attempt at surgical repair in 2014 via medial capsulorrhaphy with ADM placement. She elected to pursue another repair method in 2017 and underwent anterior capsulectomy with sub-pectoral implant removal and placement of new implants in the pre-pectoral space. ADM was utilized for soft tissue reinforcement. The pre-sternal skin was too thin to attempt anchoring with suture. Alternatively, a standard black foam negative pressure wound device was placed externally over a layer of Tegaderm to evenly compress the entire intermammary sulcus (Fig. 1) for 15 days postoperatively.

The second patient, a 54-year-old female, developed symmastia following bilateral nipple sparing mastectomy with staged sub-pectoral implant reconstruction in 2013 for ductal carcinoma in-situ of the left breast. Initial capsulorrhaphy with ADM was attempted 4 months later and failed to correct the symmastia. She was taken back in 2018 for revision, during which the prior implants were removed and the sub-pectoral capsules were excised entirely. New implants were placed in the pre-pectoral space with application of an external negative pressure wound device after closure in the same fashion for 17 days.

Lastly, the third patient is a 69-year-old female who underwent bilateral mastectomy for lobular carcinoma in-situ in 1990 and had subsequent sub-pectoral implant reconstruction. She came to our clinic via referral nearly 31 years after her index procedure with MRI evidence of bilateral implant rupture and symmastia. She underwent implant removal with thorough washout, capsulectomy, and sub-pectoral space closure. New implants were similarly placed with ADM into a new pre-pectoral plane. External compression was applied to the intermammary sulcus with an external negative pressure device for 19 days.

Medial intermammary confluence was absent after device removal in all 3 patients. Mean duration of negative pressure therapy was 17(±2) days. No complications were reported.

## Discussion

While not a threat to the overall physical health of the patient or function of the breast or surrounding tissues, symmastia is an unfortunate potential complication that compromises cosmetic outcomes following breast reconstruction and augmentation. There are several proposed mechanisms as to the cause of symmastia, each involving some element of over-dissection of medial structures or oversized implants [6, 7]. An additional proposed etiology of iatrogenic symmastia is the subsequent alteration of force vectors from the pectoralis major muscle following implant placement in the sub-pectoral plane, leading to displacement of the implants medially [7].

Correction of symmastia may be approached in several ways [2–5]. Due to high recurrence rates associated with these techniques, patients typically require more than one operation to resolve their symmastia [7].

Alternatively, several of the methods may be applied in one operation to bolster the overall repair [8]. In a recent systematic review comparing capsulorrhaphy, dermo-sternal adhesion, neo-pocket creation, muscle repair techniques, and subgroups of each of these four major categories, a global recurrence rate of 8.5% was noted, and no specific technique demonstrated superiority [9]. Of the listed techniques, capsulorrhaphy appears to have the highest recurrence rates, though these rates may be improved with the use of acellular dermal matrix [9].

In this brief case report series, all three patients demonstrated resolution of medial intermammary confluence following symmastia repair with subsequent negative pressure wound device placement (Fig. 1), indicating successful correction of symmastia. Though further research is needed to support the practice empirically, negative pressure therapy is easily accessible and noninvasive. As such, we believe this technique to be a useful adjunctive repair strategy for mitigating recurrence of symmastia.
